# Neural substrates of mnemonic discrimination: A whole‐brain fMRI investigation

**DOI:** 10.1002/brb3.1560

**Published:** 2020-02-03

**Authors:** Jenna L. Klippenstein, Shauna M. Stark, Craig E. L. Stark, Ilana J. Bennett

**Affiliations:** ^1^ Department of Psychology University of California Riverside California; ^2^ Department of Neurobiology & Behavior University of California Irvine California

**Keywords:** cortex, episodic memory, fMRI, hippocampus, mnemonic discrimination

## Abstract

**Introduction:**

A fundamental component of episodic memory is the ability to differentiate new and highly similar events from previously encountered events. Numerous functional magnetic resonance imaging (fMRI) studies have identified hippocampal involvement in this type of mnemonic discrimination (MD), but few studies have assessed MD‐related activity in regions beyond the hippocampus. Therefore, the current fMRI study examined whole‐brain activity in healthy young adults during successful discrimination of the test phase of the Mnemonic Similarity Task.

**Method:**

In the study phase, participants made “indoor”/“outdoor” judgments to a series of objects. In the test phase, they made “old”/“new” judgments to a series of probe objects that were either repetitions from the memory set (targets), similar to objects in the memory set (lures), or novel. We assessed hippocampal and whole‐brain activity consistent with MD using a step function to identify where activity to targets differed from activity to lures with varying degrees of similarity to targets (high, low), responding to them as if they were novel.

**Results:**

Results revealed that the hippocampus and occipital cortex exhibited differential activity to repeated stimuli relative to even highly similar stimuli, but only hippocampal activity predicted discrimination performance.

**Conclusions:**

These findings are consistent with the notion that successful MD is supported by the hippocampus, with auxiliary processes supported by cortex (e.g., perceptual discrimination).

## Graphical abstract

Mnemonic discrimination is a fundamental component of episodic memory that has typically been attributed to the hippocampus, with little attention paid to regions beyond the medial temporal lobe. Here, we examined neural signatures of mnemonic discrimination across the brain. Results revealed significant activity in hippocampus and occipital cortex, but only hippocampal activity predicted mnemonic discrimination performance.

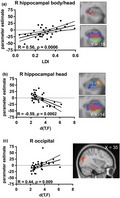

## 1 INTRODUCTION

Mnemonic discrimination (MD) is an essential component of episodic memory that allows for the differentiation of new stimuli relative to previously encountered stimuli, even when they are highly similar (e.g., the breakfast you ate today vs. yesterday). Using modified recognition memory paradigms, such as the Mnemonic Similarity Task (MST; Stark, Yassa, Lacy, & Stark, 2013), MD is seen as differentially endorsing lures that are similar to, but not the same as, previously presented targets (i.e., judging lures as “new” instead of “old”). In study/test recognition paradigms, lures only appear in the separate test phase as stimuli similar to those presented during the study phase (Huffman & Stark, 2017; Stark, Stevenson, Wu, Rutledge, & Stark, 2015). In continuous recognition paradigms, similar lures and repeated targets are presented in a series with no intervening delay (Bakker et al., 2012; Kirwan & Stark, 2007). For both paradigms, the degree of similarity between lures and targets can be parametrically manipulated, resulting in worse discrimination performance as lure similarity increases (Lacy, Yassa, Stark, Muftuler, & Stark, 2011; Motley & Kirwan, 2012).

Numerous functional magnetic resonance imaging (fMRI) studies have identified MD‐related activity in the hippocampus, consistent with its proposed role in the computational process that supports our ability to differentiate between stimuli (i.e., pattern separation; Yassa & Stark, 2011). Most studies have used a repetition sensitivity approach (see Kim, 2017 for a review), in which regions sensitive to repetition are first identified by comparing activity to repeated and novel stimuli. Within these repetition‐sensitive regions, the neural signature of successful MD is observed as activity to lures that is different from repeated targets and similar to novel stimuli, which has been observed in hippocampus (Berron et al., 2018) and its subfields, specifically the dentate gyrus/cornu ammonis 3 (DG/CA3; Azab, Stark, & Stark, 2014; Bakker, Kirwan, Miller, & Stark, 2008; Kirwan & Stark, 2007; Lacy et al., 2011). In some cases, a lure‐similarity approach has been used in conjunction with the repetition sensitivity approach by first identifying regions sensitive to repetition and then testing whether activity to lures that parametrically vary in their degree of similarity to targets is significantly different from repeated targets but not novel stimuli (Lacy et al., 2011). For example, one study found that repetition‐sensitive regions in hippocampus and parahippocampal cortex were best fit by a power function modeling the difference in activity between repeated targets and lures that parametrically varied across four levels of lure similarity (Motley & Kirwan, 2012). To our knowledge, these hippocampal effects have not been assessed using study/test recognition paradigms for which the repetition sensitivity approach may be less sensitive to successful MD when initial and subsequent presentations of repeated stimuli occur in separate task phases separated by a long interval.

In addition to medial temporal regions, growing fMRI evidence suggests that neocortical regions also exhibit activity consistent with MD (Motley & Kirwan, 2012; Reagh et al., 2018; Wais, Jahanikia, Steiner, Stark, & Gazzaley, 2017). For example, one previous study examined MD‐related activity across the whole brain, comparing targets and related items based on perceptual and conceptual similarity (Pidgeon & Morcom, 2016). Using a repetition sensitivity approach, prefrontal and occipitotemporal cortices revealed greater activity to related and non‐related items relative to targets (comparable to similar lures = novel stimuli > repeated targets). Using a lure‐similarity approach within regions sensitive to repetition, they further observed MD‐related activity in inferior frontal and supramarginal gyri with a power function that modeled the difference in activity to repeated targets relative to lures that parametrically varied across three levels of lure similarity. Importantly, their incidental study phase presented non‐related (novel), repeated targets, and related (lure) items in a series, comparable to a continuous paradigm. Thus, as in the hippocampal literature, testing these cortical effects using a study/test recognition paradigm with longer intervals between initial and subsequent presentations of repeated and lure stimuli may better isolate cortical regions that support successful MD from related processes, such as perceptual discrimination.

In contrast to empirical findings of cortical activity consistent with MD, there is minimal theoretical support for direct cortical involvement in MD. Multiple theoretical accounts attribute pattern separation to hippocampus (specifically the DG subfield), with limited roles for adjacent medial temporal regions (e.g., memory reinstatement, learning statistical regularities, visual feature extraction; Norman, 2010; O'Reilly, Bhattacharyya, Howard, & Ketz, 2014; Rolls, 2016). Because cortical neurons exhibit slower learning rates and overlapping activation patterns (Atallah, Frank, & O'Reilly, 2004), it is possible that regions beyond hippocampus cannot support the rapid MD of highly similar stimuli. Instead, previous fMRI studies finding MD‐related activity in cortex may have been biased by capitalizing on the repetition sensitivity approach. In addition to identifying regions sensitive to repetition, a similar contrast is also used to assess traditional recognition memory (i.e., hits vs. correct rejections; Kim, 2013). Thus, rather than detecting the neural substrates of MD, these patterns may reflect cortical regions involved in more general recognition or perceptual processes. This may be especially true when averaging across lure‐similarity conditions as it may obscure the critical difference between repeated targets and highly similar lures.

Therefore, the present study assessed hippocampal and whole‐brain MD‐related activity while young adults performed a study/test version of the MST. Activity consistent with MD was assessed during the test phase using a lure‐similarity approach sensitive to differences between targets and highly similar lures without being constrained by repetition sensitivity. First, we aimed to replicate and extend findings of MD‐related activity in the hippocampus. Then, we explored whether similar patterns could be observed when the same contrast was applied to the whole brain. Finally, we looked at the relationship between neural activity and MST performance. We hypothesized that if MD is a process that extends beyond the hippocampus, then we should observe patterns consistent with MD across neocortical regions previously implicated in MD (e.g., prefrontal, medial temporal, supramarginal, and occipitotemporal regions) using our relatively more stringent approach (step function, study/test design) and that this activity should relate to better discrimination performance. If instead, previous reports of cortical involvement in MD were due to methodological differences (such as the confound with perceptual discrimination), then we may not observe cortical effects here, which would be consistent with theoretical accounts of MD.

## 2 METHOD

### 2.1 Participants

Forty‐nine healthy young adults were recruited from the undergraduate research pool at the University of California, Riverside. Fifteen participants were excluded: poor general cognition (*n* = 3; i.e., <27 on the Mini‐Mental State Exam, MMSE; Folstein, Folstein, & McHugh, 1975), task‐related issues in the scanner (*n* = 1; e.g., stimuli presentation program failed), and poor MST performance (*n* = 11; e.g., responding “new” to every trial, <40 missed responses, or traditional recognition memory scores at or below chance). The final sample was 34 individuals (mean age = 20.07 ± 1.80; 14 females; 32 right‐handed; mean years of education = 12.68 ± 1.01).

Prior to enrollment in the study, participants were screened for conditions that would affect their ability to complete the computer‐based task (e.g., uncorrectable vision), prevent them from being able to enter the MRI scanner (e.g., pregnancy, non‐MR compliant implants, difficulty lying in the supine position, or claustrophobia), or impair their cognitive functioning (e.g., stroke, diabetes, or uncontrolled depression). All study procedures were conducted in compliance with the Institutional Review Board (IRB) of the University of California, Riverside, and all participants provided informed consent and received course credit for participation.

### 2.2 Mnemonic Similarity Task

**Figure 1:**
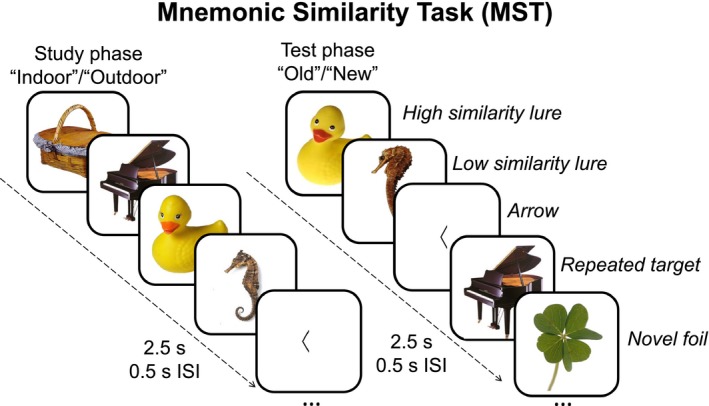
The Mnemonic Similarity Task (MST) used here consisted of an incidental study phase followed by a test phase. During the study phase, participants made “indoor”/“outdoor” judgments to a series of objects. During the test phase, participants made “old”/“new” judgments to novel foils, high‐ or low‐similarity lures, and repeated targets. For both phases, intermittent left‐ or right‐facing arrows were presented, and participants were asked to judge the respective direction

During fMRI scanning, participants completed an incidental study phase  followed by a test phase of the MST (Stark et al., 2013; Figure 1). In the study phase, participants made “indoor” or “outdoor” judgments to a series of 128 common objects via a left‐ and right‐handed button press, respectively. In the test phase, participants made “old” or “new” judgments to a series of 192 probe objects that were either exact repetitions of objects presented in the study phase (64 repeated targets), similar to objects presented in the study phase (64 similar lures), or novel first presentations (64 novel foils). Lures were divided into five bins based on their similarity to targets where lure bin 1 represents the highest similarity between the studied and test items and lure bin 5 represents the lowest similarity (12–13 lures per bin; see Lacy et al., 2011 for details).

Of note, compared to previous fMRI MST studies that have employed a 3‐choice response format (“old,” “similar,” “new”), the 2‐choice format used here removes ambiguity related to the “similar” response (e.g., individual differences in the threshold for responding to lures as “similar” vs. “new”), which has advantages for our examination of the behavioral and neural correlates of MD, including the use of signal detection theory. Previous studies employing this 2‐choice response format have revealed similar behavioral performance to the 3‐choice format when participants are instructed to respond to any object that is not exactly the same as something they previously saw as “new,” as was done here (Leal, Noche, Murray, & Yassa, 2017; Stark et al., 2015; Wais et al., 2017).

Participants also completed an arrows task that was randomly interspersed throughout the MST trials and served as a non‐mnemonic baseline condition that minimally engages the hippocampus (Stark & Squire, 2001). On these 20 trials, a left‐ or right‐facing arrow was presented, and participants made “left” or “right” judgments via a button press, respectively. “Left” and “old” responses were made with the left hand, and “right” and “new” responses were made with the right hand. All stimuli for the MST (color objects) and arrows task (black arrow) were presented on a white background for 2.5 s with a 0.5‐s inter‐stimulus interval.

Traditional recognition memory (recognition) was measured as the probability of correctly judging a repeated target as “old” minus the probability of incorrectly judging a novel foil as “old” (i.e., hits minus false alarms). Recognition memory was also measured using a *d*′ score (*d*[T, F]), which is used in signal detection theory to provide a separation between the means of the signal and noise distributions to mitigate the effect of any response bias. The recognition *d*′ score was calculated as the difference in the distributions between “old” responses to targets and “old” responses to foils (Stark et al., 2015).

Consistent with previous work (Wais et al., 2017), MD was measured using a lure discrimination index (LDI), calculated as the proportion of “new” responses to lures minus the proportion of “new” responses to targets. Mnemonic discrimination was also measured using a *d*′ score (*d*[T, L]), calculated as the difference in the distributions between “old” responses to targets and “old” responses to lures (Stark et al., 2015). In addition, separate high‐ and low‐similarity *d*′ scores were calculated as the difference in distributions between “old” responses to targets and “old” responses to either high (lure bins 1 and 2)‐ or low (lure bins 3, 4, and 5)‐similarity lures, respectively.

### 2.3 Imaging data acquisition

Imaging data were collected at the Center for Advanced Neuroimaging at the University of California, Riverside, on a 3 Tesla Siemens Prisma MRI scanner (Siemens Medical Solutions) equipped with a 32‐channel receive‐only head coil. A single T1‐weighted magnetization prepared rapid acquisition gradient echo (MP‐RAGE) sequence was acquired with the following parameters: repetition time (TR)/echo time (TE) = 2,400/2.72 ms, field of view = 256 × 256 × 208 mm, flip angle = 8 degrees, and spatial resolution = 0.8 mm^3^.

A single echo‐planar imaging (EPI) pulse sequence was acquired during performance of the MST test phase with the following parameters: TR/TE = 1,750/32 ms, field of view = 221 × 190.4 mm, flip angle = 75 degrees, spatial resolution = 1.7 mm^3^, 72 slices with no gap, AP phase‐encoding direction, GRAPPA acceleration factor = 2, and multiband factor = 3.

Additional sequences were acquired to correct for susceptibility distortions in each participant's functional data. For the first six participants, phase maps from a dual gradient echo pulse sequence were acquired with the following parameters: TR/TE_1_/TE_2_ = 662/4.92/7.38 ms, spatial resolution = 2 mm^3^, and 68 slices. For remaining participants, two sets of spin‐echo EPI images with phase‐encoding directions of opposite polarity were acquired using parameters identical to the EPI sequence in the functional run, except TR/TE = 7,700/58 ms.

### 2.4 Functional imaging data analysis

#### 2.4.1 Preprocessing

Functional imaging data were analyzed with FSL (FMRIB's Software Library, http://www.fmrib.ox.ac.uk/fsl). For each participant, raw functional data were corrected for susceptibility‐induced distortions using either the field map method implemented in FUGUE (FMRIB's Utility for Geometrically Unwarping EPIs) or the blip‐up blip‐down method as implemented in FSL (Andersson, Skare, & Ashburner, 2003). Distortion corrected images were then subjected to the following preprocessing steps in FEAT (FMRI Expert Analysis Tool): skull stripping using the brain extraction tool (Smith, 2002), spatial smoothing using a Gaussian kernel with a full width at half maximum of either 3 mm (for hippocampal region of interest analyses) or 5 mm (for whole‐brain analyses), and high‐pass filtering (100 s). Data were then registered to the participant's T1‐weighted image using FLIRT (FMRIB's Linear Image Registration Tool; Jenkinson, Bannister, Brady, & Smith, 2002; Jenkinson & Smith, 2001) and then to the standard Montreal Neurological Institute (MNI) 152 template (resampled to 1.7‐mm^3^ resolution) using a combination of FLIRT and FNIRT (FMRIB's Nonlinear Image Registration Tool; Andersson, Jenkinson, Smith, & Andersson, 2007). Finally, data were de‐noised using probabilistic independent component analysis (Beckmann & Smith, 2004) as implemented in FSL's MELODIC (Multivariate Exploratory Linear Decomposition into Independent Components). A trained researcher manually selected noisy components to be regressed out from preprocessed data. The final de‐noised data were used as input for the lower‐level analyses.

#### 2.4.2 Analyses

Mnemonic discrimination‐related activity was assessed using voxel‐wise step function and correlational approaches within a hippocampal region of interest and across the whole brain as described below. For the former, analyses were limited to an anatomical mask of bilateral hippocampus (>40% probability in FSL's Harvard‐Oxford subcortical atlas). For the latter, analyses were unconstrained anatomically and included the hippocampus. Lower‐level analysis explanatory variables (EVs) were convolved with a gamma‐variate hemodynamic response function (standard deviation = 3 s, mean lag = 6 s). Higher‐level analyses used FSL's Local Analysis of Mixed Effects (FLAME) stage 1. Significant clusters were identified using either a liberal threshold consistent with prior work for the hippocampal analyses (uncorrected cluster thresholding of *p* < .05, ≥20 contiguous voxels; Lacy et al., 2011) or corrected cluster thresholding for the whole‐brain analyses with false discovery rate procedures to correct for multiple comparisons (*z* > 2.7, *p* < .05).

For all analyses, a lower‐level analysis for each participant separately modeled “old” and “new” responses to each MST trial type (high [lure bins 1 and 2]‐ and low [lure bins 3, 4, and 5]‐similarity lures, targets, foils), “left” and “right” responses to arrows, and no response trials, yielding a total of eleven EVs. Separate contrasts captured mean effects for each MST trial type relative to the response‐matched arrow condition (e.g., “new”|foil > “right”|arrow).

For the voxel‐wise step function analysis, separate mid‐level analyses first estimated step function activity for each participant. Using baseline‐corrected inputs from the lower‐level contrasts, one EV modeled correct responses to targets, high‐similarity lures, low‐similarity lures, and foils using weights of −3, 1, 1, and 1, respectively (averaging 58.0, 18.7, 6.2, and 57.2 trials per participant, respectively). A bidirectional (two‐tailed) contrast assessed where activity to targets was greater or less than average activity to the other three trial types (i.e., “old”|target vs. “new”|high‐similarity lure = “new”|low‐similarity lure = “new”|foil). A higher‐level analysis then assessed the mean of this step function across the group using one EV to model each participant's mean step function from the mid‐level analysis and a bidirectional (two‐tailed) contrast to assess the step function as above.

For the voxel‐wise correlations, separate higher‐level analyses were conducted between MD‐related activity and each measure of MD performance (LDI, *d*′[T, L]) as well as each measure of recognition memory (recognition, *d*′[T, F]). For each behavioral measure, one EV modeled each participant's mean step function activity from the mid‐level analysis and a second EV modeled each participant's performance. A bidirectional (two‐tailed) contrast identified regions where step function activity positively or negatively correlated with performance.

For the hippocampus, we reported the location of significant clusters by delineating hippocampal head, body, and tail at *y* = −20 and −35 in MNI space, in line with prior work (DeMaster & Ghetti, 2013; Sastre, Wendelken, Lee, Bunge, & Ghetti, 2016), to inform literature suggesting functional specialization along the longitudinal axis of the hippocampus (Hrybouski et al., 2019). We also overlaid significant clusters on a standard hippocampal subfield template (Stark & Stark, 2017).

## 3 RESULTS

### 3.1 Mnemonic discrimination performance

Recognition memory was assessed with a one‐sample, two‐tailed *t *test comparing traditional MST recognition performance and *d*′ to chance (0.5) and zero, respectively. A significant effect indicated that participants accurately distinguished novel foils from repeated targets using the traditional recognition memory (0.84 ± 0.09), *t*(33) = 22.55, *p* < .0001, and *d*′(T, F) (1.13 ± 0.55), *t*(33) = 19.03, *p* < .0001, metrics.

Mnemonic discrimination was assessed with a one‐sample, two‐tailed *t *test comparing MD performance to zero. A significant effect indicated that participants were sensitive to differences between similar lures and repeated targets using LDI (0.30 ± 0.07), *t*(33) = 13.89, *p* < .0001, and *d*′(T, L) (1.13 ± 0.55), *t*(33) = 11.87, *p* < .0001, metrics.

Mnemonic discrimination performance was further assessed using a paired, two‐tailed *t *test to compare *d*′ scores between lure‐similarity conditions. As expected, results revealed significantly worse MD performance on the more difficult high (*d*′ = 0.68 ± 0.57)‐similarity compared to low (*d*′ = 1.57 ± 0.68)‐similarity lures, *t*(33) = 11.86, *p* < .0001.

#### 3.1.1 Step function

**Figure 2:**
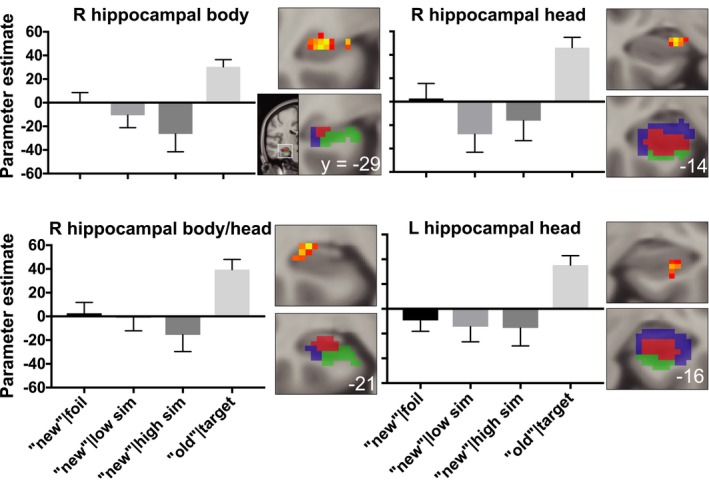
Four hippocampal clusters identified in the voxel‐wise step function analysis are displayed in red‐yellow on coronal slices (top right of each bar graph). Displayed results were thresholded uncorrected (*p* < .05, ≥20 contiguous voxels), presented in Montreal Neurological Institute (MNI) 152 space, and in radiological orientation (right = left). For reference, a hippocampal subfield template identified using a multi‐atlas model (see Stark & Stark, 2017 for details) that was aligned to Montreal Neurological Institute (MNI) 152 space displays DG/CA3 (red), CA1 (blue), and subiculum (green) subfields on coronal slices (bottom right of each bar graph). For illustrative purposes, bar graphs display the parameter estimates from each hippocampal cluster identified from the voxel‐wise analysis. Of note, the directionality of the bars in this Figure is inconsequential because the direction of fMRI activity is dependent on multiple elements of the design, including the specific trials being contrasted, the frequency of those conditions, and which conditions are in the model. Thus, we assess step function patterns independent of the direction of the difference. Consistent with mnemonic discrimination, the average activity to correct “new” responses to foils and low‐ and high‐similarity lures differs from activity to correct “old” responses to targets. Error bars display standard error of the mean

Using two separate voxel‐wise step functions for hippocampus and the whole brain, we tested whether activity to lures was significantly different from repeated targets but not novel stimuli. When limited to an anatomical mask of the bilateral hippocampus, this approach revealed four significant clusters in the bilateral body, right head, and right body/head of the hippocampus, all of which show overlap with the DG/CA3 (Table 1). For illustrative purposes, the parameter estimates from these significant clusters are displayed in Figure 2.

**Table 1:** Hippocampal mean and mnemonic discrimination effects

Effect	Region	*x*	*y*	*z*	*Z*‐max	Voxels
Targets mean effect	R head	20	−12	−18	3.25	72
	R body	29	−29	−7	3.77	61
	R head	−27	−5	−24	2.61	33
	L head	37	−19	−14	3.22	26
	L head	−21	−14	−16	2.82	23
Low‐similarity lures mean effect	L body/tail	−29	−34	−6	2.29	21
	L head	−29	−19	−16	2.39	20
Foils mean effect	R head	31	−12	−19	2.84	64
	L head	−32	−17	−16	2.96	55
	L body	31	−22	−19	2.79	24
MD step function	R body	31	−29	−9	3.38	47
	R head	20	−10	−19	2.73	40
	L head	−29	−10	−24	2.71	34
	R body/head	34	−21	−11	2.88	27

Note

Hippocampal clusters showing significant mean and mnemonic discrimination‐related effects are described with their peak voxel (*x*, *y*, *z* coordinates in Montreal Neurological Institute 152 space), corresponding maximum *z*‐statistic (*Z*‐max), and spatial extent (number of voxels).

Abbreviations: L, left hemisphere; R, right hemisphere.

**Figure 3:**
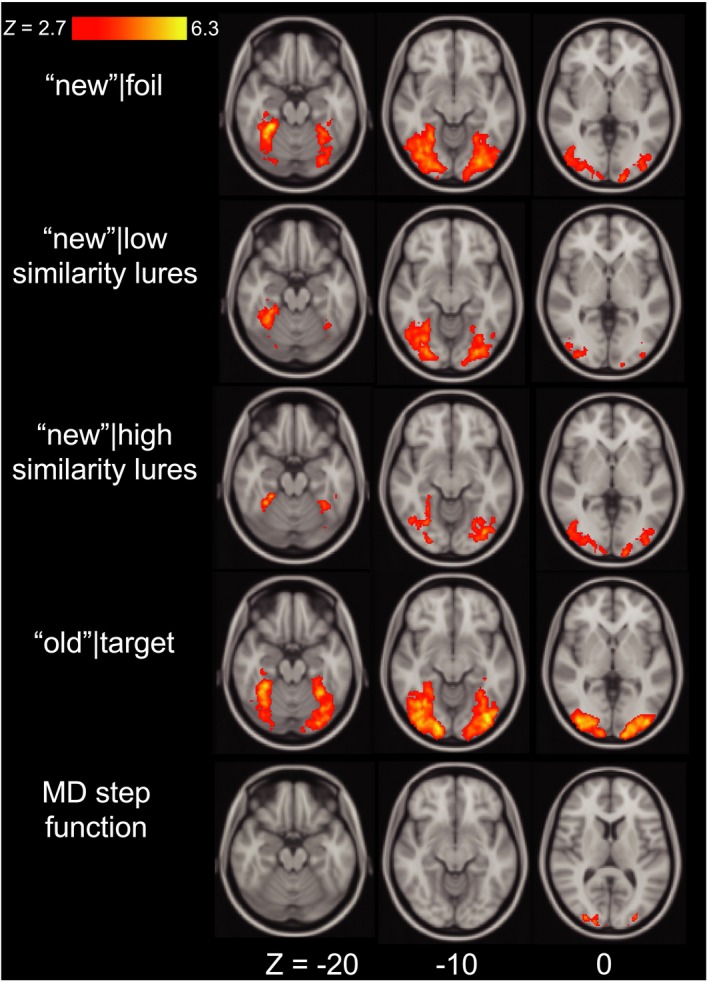
Whole‐brain clusters exhibiting significant mean and mnemonic discrimination effects are displayed on axial slices. Displayed results were cluster extent corrected at *Z* > 2.7, *p* < .05, presented in Montreal Neurological Institute (MNI) 152 space and in radiological orientation (right = left)

**Figure 4:**
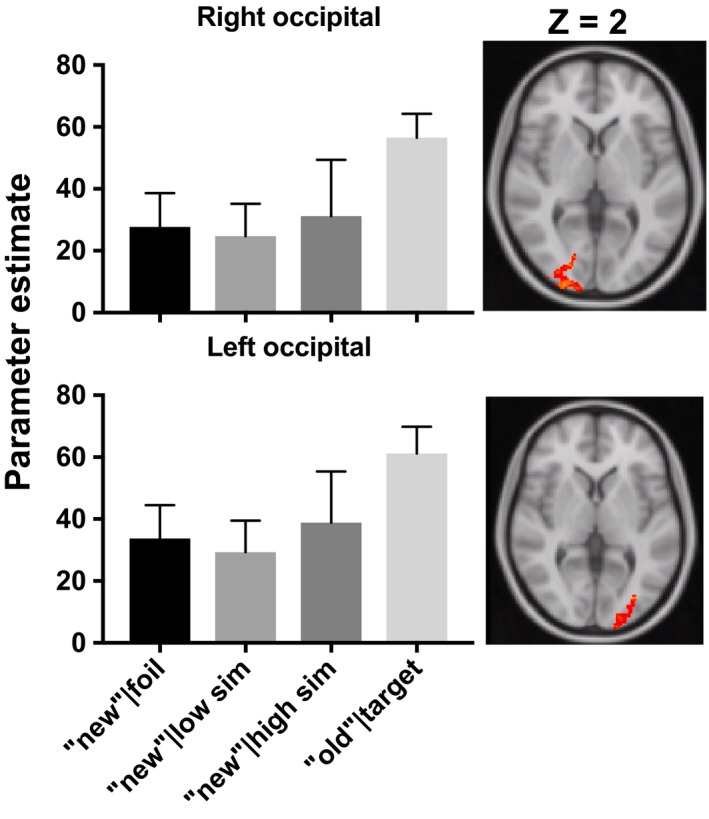
Two occipital clusters identified in the voxel‐wise step function analysis are displayed in red‐yellow on axial slices (right of each bar graph). Displayed results were cluster extent corrected at *Z* > 2.7, *p* < .05, presented in Montreal Neurological Institute (MNI) 152 space and in radiological orientation (right = left). For illustrative purposes, bar graphs display the parameter estimates from each occipital cluster identified from the voxel‐wise analysis. Consistent with mnemonic discrimination, the average activity to correct “new” responses to foils and low‐ and high‐similarity lures differs from activity to correct “old” responses to targets. Error bars display standard error of the mean

When applied to the whole brain, this approach revealed significant activity in bilateral occipital clusters. These results are shown in Figures 3 and 4 and Table 2.

**Table 2:** Whole‐brain mean and mnemonic discrimination effects

Effect	Region	*x*	*y*	*z*	*Z*‐max	Voxels
Targets mean effect	R occipitotemporal	17	−92	−9	6.06	7,978
	L occipitotemporal	−39	−80	−11	6.12	6,951
High‐similarity lures mean effect	L occipitotemporal	−29	−56	−16	4.44	1,315
	R occipitotemporal	32	−48	−18	4.84	1,144
Low‐similarity lures mean effect	R occipitotemporal	27	−50	−14	5.54	3,454
	L occipitotemporal	−32	−67	−13	5.05	1,941
Foils mean effect	R occipitotemporal	27	−51	−14	6.26	6,115
	L occipitotemporal	−31	−58	−16	5.98	4,832
MD step function	R occipital	27	−94	−2	3.66	692
	L occipital	−29	−84	−1	3.77	351

Note

Whole‐brain clusters showing significant mean and mnemonic discrimination‐related effects are described with their peak voxel (*x*, *y*, *z* coordinates in Montreal Neurological Institute 152 space), corresponding maximum *z*‐statistic (*Z*‐max), and spatial extent (number of voxels).

Abbreviations: L, left hemisphere; R, right hemisphere.

#### 3.1.2 Voxel‐wise correlations

**Figure 5:**
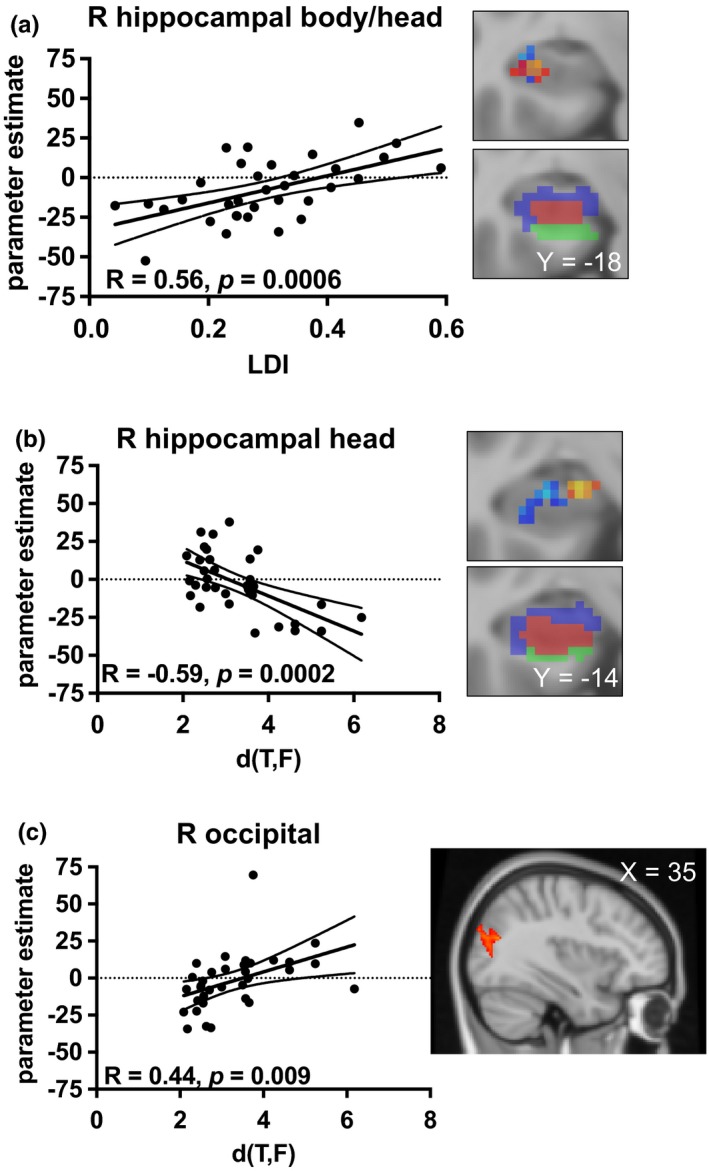
Scatterplots display relationships between MST performance and parameter estimates from step function activity. For (a) and (b), clusters from the step function analysis and voxel‐wise correlations are shown in red‐yellow and in blue, respectively. Significant relationships were seen between mnemonic discrimination (lure discrimination index, LDI) and activity in right hippocampal body/head (a), and between recognition performance (*d*′[T,F]) and activity in right hippocampal head (b) and right occipital cortex (c). Dashed lines represent 95% confidence intervals

Using two separate voxel‐wise correlations for hippocampus and the whole brain, we examined whether behavioral discrimination of similar lures (LDI, *d*′[T, L]) related to step function activity. When limited to an anatomical mask of the bilateral hippocampus, we found a significant positive correlation between LDI and step function activity in one cluster that largely overlapped with the right hippocampal body/head cluster identified from the previously reported step function analysis (Figure 5a). In contrast, when this analysis was expanded to the whole brain, there was no significant relationship between either behavioral measure of MD (LDI, *d*′[T, L]) and whole‐brain step function activity in any region.

Using two separate voxel‐wise correlations for hippocampus and the whole brain, we also examined whether traditional recognition memory (recognition, *d*′[T, F]) related to step function activity. When limited to an anatomical mask of the entire hippocampus, we found a significant negative correlation between *d*′(T, F) and step function activity in a right hippocampal head cluster that was primarily located within CA1 and did not overlap with any cluster from the voxel‐wise step function analysis (Figure 5b). When applied to the whole brain, we found a significant positive correlation between *d*′(T, F) and step function activity in the right occipital cortex, although this cluster was located more superior than the cluster identified in the voxel‐wise step function analysis (Figure 5c). There were no significant relationships between traditional recognition memory scores and hippocampal or whole‐brain step function activity.

## 4 DISCUSSION

The present study separately examined hippocampal and brain‐wide MD‐related activity in young adults during the test phase of the MST. Relative to previous work, we used a lure‐similarity analysis that was sensitive to differences between targets and highly similar lures without being constrained by repetition sensitivity. We employed a study/test design and 2‐choice response format to ensure that effects are primarily attributed to discrimination of mnemonic representations that occurs at test. Results revealed significant neural activity consistent with MD in the bilateral hippocampus, consistent with previous findings. In addition, an exploratory whole‐brain analysis revealed a similar pattern of activity in the occipital cortex. However, only activity in the hippocampus related to MD performance, emphasizing the role of the hippocampus in discriminating between highly similar experiences.

Mnemonic discrimination is defined as the ability to differentiate between new and previously encountered stimuli, even when they are highly similar. Thus, we fit a step function to the imaging data to identify where the neural response to both high‐ and low‐similarity lures was comparable to novel foils but different from repeated targets. Results revealed four significant clusters in bilateral hippocampus. A similar pattern was also reported in bilateral hippocampus in a previous study that fits a power function to the neural response to targets and lures with four degrees of similarity (Motley & Kirwan, 2012). This study, like others that have demonstrated MD‐related activity in hippocampus (Bakker et al., 2008; Kirwan & Stark, 2007; Lacy et al., 2011; Yassa, Mattfeld, Stark, & Stark, 2011), used a continuous recognition paradigm and limited their curve fitting analysis to regions first identified using a repetition‐sensitive approach. Here, we extend this literature by replicating the findings using a lure‐similarity approach on test phase data from a study/test paradigm, providing confidence that significant effects in hippocampus are capturing the neural substrates of MD, rather than other processes that may underlie repetition sensitivity (e.g., sensitivity to repetition, more general recognition memory, and novelty detection).

Both the MD‐related activity in the hippocampus and its positive relationship with lure discrimination performance are consistent with the notion that hippocampus is directly involved in the computational process that supports MD (i.e., pattern separation; Yassa & Stark, 2011). The DG subfield of the hippocampus may be uniquely structured to support orthogonal representations, even between highly similar stimuli (Norman, 2010; O'Reilly et al., 2014; Rolls, 2016). High‐resolution (1.5 mm^3^) fMRI studies have observed (but not explicitly tested) a stepwise transfer function in DG/CA3, showing activity to repeated targets that differs from similar lures and novel foils (Lacy et al., 2011; Yassa et al., 2011), prompting our use of the step function tested here. Of note, in spite of the lower spatial resolution (1.7 mm^3^) used here, our step function clusters appear to primarily overlap with DG/CA3 (Figure 2). These relatively more posterior clusters are also consistent with the notion that posterior hippocampus is involved in detailed episodic memories (Hrybouski et al., 2019; Poppenk, Evensmoen, Moscovitch, & Nadel, 2013; Strange, Witter, Lein, & Moser, 2014), which is necessary for successful MD.

We also found that bilateral occipital cortex was sensitive to MD‐related activity, but that MD‐related activity in an adjacent right occipital cluster was significantly related to recognition memory, not lure discrimination performance. Previous studies have observed similar engagement of occipital cortex during MD using continuous recognition paradigms and repetition‐sensitive analysis approaches (Motley & Kirwan, 2012; Pidgeon & Morcom, 2016). Functional connectivity between the hippocampus and early visual regions during MD (Paleja, Girard, Herdman, & Christensen, 2014) further suggests that cortico‐hippocampal interactions support discrimination performance. However, rather than playing a direct role in MD, theoretical accounts propose that occipital cortex contributes to memory recollection by providing a visual representation for the hippocampus to pattern separate (Rolls, 2017). Because cortical activity was not related to discrimination performance, our findings support the notion that cortex contributes, but is not directly involved, in MD.

Within the hippocampus, our voxel‐wise correlation approach revealed that step function activity was significantly related to both LDI and *d*′(T, F). The relationship between step function activity and LDI, but not between step function activity and *d*′(T, F), overlapped with significant clusters from the voxel‐wise step function analysis. Finding that LDI performance correlated with MD‐related activity in regions that showed suprathreshold step function activity provides strong support for our hypothesis (as well as theoretical accounts) that the hippocampus is directly involved in MD. Finding that recognition performance correlated with MD‐related activity in clusters that were distinct from those related to LDI is consistent with the notion that different hippocampal subregions mediate different mnemonic processes. Importantly, recognition performance correlated with activity in regions that did not show suprathreshold step function activity. Thus, relationships with recognition performance may be driven by our step function modeling the difference between targets and foils (a distinction that is needed to support better recognition) in regions that are not sensitive to the difference between targets and lures (consistent with MD).

Cortex likely supports other related processes, such as perceptual discrimination. For example, with continuous recognition paradigms that have a relatively short duration between the initial presentation of a stimulus and its subsequent presentation as a repeated target or similar lure (e.g., 1.5–3 min; Pidgeon & Morcom, 2016), new and previously encountered stimuli may be differentiated based on stimulus features (e.g., perceptual discrimination). Our use of a study/test recognition paradigm that had at least 7 min between the initial and subsequent presentations was intended to increase the likelihood that participants had to rely on their memory of the initial presentation in order to make the correct mnemonic judgment at test, biasing our results to reflect MD. Nonetheless, there are still perceptual discrimination aspects to our task (e.g., comparing a presented lure to the memory trace of the target). Although our current study design does not allow us to parametrically assess perceptual similarity, one interpretation of our voxel‐wise step function finding in occipital cortex is that the contrast may be sensitive to both mnemonic and perceptual discrimination.

Of note, one other study previously assessed MD‐related activity during the test phase of a study/test recognition task (Wais et al., 2017), however, using a different fMRI contrast. Mnemonic discrimination was defined as greater activity to accurately versus inaccurately discriminated lures (responding “new” vs. “old”), which was observed in a priori medial temporal (hippocampus, entorhinal) and cortical (inferior frontal, lateral parietal) regions of interest. In light of evidence that the hippocampus can distinguish between previously encountered and new stimuli independent of their behavioral response (Daselaar, Fleck, Prince, & Cabeza, 2006; Kirwan, Shrager, & Squire, 2009), it is more likely that this contrast is capturing regions involved in a subjective sense of lure “oldness” or setting an “old”/“new” response criteria. Regardless, it does not consider the neural responses to lures relative to targets, which we argue should be the hallmark of any contrast that defines successful MD as the ability to differentiate between previously encountered and new stimuli. The present lure‐similarity approach, which adopts a more conservative interpretation of successful MD that requires differentiating between previously encountered and highly similar new stimuli, provides a novel and potentially more accurate assessment of MD‐related activity.

In summary, this study is the first to assess hippocampal and whole‐brain MD‐related activity during the test phase of a study/test recognition paradigm using a lure‐similarity approach alone. Results revealed that the hippocampus and occipital cortex exhibited differential activity to repeated stimuli relative to even highly similar stimuli, but only hippocampal activity predicted MD performance. These findings are consistent with the notion that MD is limited to the hippocampus, whereas cortex may be more involved in related processes such as general recognition or perceptual discrimination.

## CONFLICT OF INTEREST

None declared.

## Data Availability

The data that support the findings of this study are available from the corresponding author upon reasonable request.
